# Demand creation for testing and Covid-19 surveillance indicators in the Covid-19 Testing, Isolation, Quarantine, and Telemonitoring Study

**DOI:** 10.11606/s1518-8787.2026060006626

**Published:** 2026-05-01

**Authors:** Diana Zeballos, Fabiane Soares, Laio Magno, Thais Aranha Rossi, Gabriel Alves de Sampaio Morais, Carina Carvalho dos Santos, Joice Neves Reis, Guilherme Barreto Campos, Lucas Miranda Marques, Alexandre Grangeiro, Debora Castanheira, Thiago Silva Torres, Valdilea Gonçalves Veloso, Ines Dourado

**Affiliations:** I Universidade Federal da Bahia. Instituto de Saúde Coletiva. Salvador, BA, Brasil; II Universidade do Estado da Bahia. Departamento de Ciências da Vida. Salvador, BA, Brasil; III Fundação Oswaldo Cruz. Instituto Gonçalo Moniz. Salvador, BA, Brasil; 4 Universidade Federal da Integração Latino Americana. Instituto Latino-Americano de Economia, Sociedade e Política. Foz do Iguaçu, PR, Brasil; V Universidade Federal da Bahia. Faculdade de Farmácia. Departamento de Análises Clínicas e Toxicológicas. Salvador, BA, Brasil; VI Universidade Federal da Bahia. Instituto Multidisciplinar em Saúde. Vitória da Conquista, BA, Brasil; VII Universidade de São Paulo. Faculdade de Medicina. São Paulo, SP, Brasil; VIII Fundação Oswaldo Cruz. Instituto Nacional de Infectologia Evandro Chagas. Rio de Janeiro, RJ, Brasil

**Keywords:** COVID-19, Public Health Surveillance, Primary Health Care, Covid-19 Testing

## Abstract

**OBJECTIVE:**

To analyze the demand creation strategies for Covid-19 testing and to describe surveillance indicators for testing, quarantine, contact tracing, and telemonitoring in primary health care services.

**METHODS:**

This cross-sectional study used data from the Covid-19 Testing, Isolation, Quarantine, and Telemonitoring (TQT Covid-19) study, conducted from July 2022 to July 2023. Primary healthcare service units in Rio de Janeiro and Salvador were selected to apply an intervention to develop demand creation strategies for testing uptake. Demand creation strategies were grouped into online strategies, traditional means, primary health care service units, community, and active search. Logistic regression was performed to determine the characteristics of the population reached by each strategy. The following Covid-19 surveillance indicators were estimated for testing and prevention (testing rate, positivity rate, monthly incidence, and full vaccination rate); for telemonitoring (monitoring rate, severity, referrals, and mortality); for quarantine (quarantine conditions); and for contact tracing (proportion of contacts traced and refusals of testing).

**RESULTS:**

The intervention reached 12,401 individuals, and 11,843 tests were performed. Demand creation strategies that reached more individuals for testing were primary health care service units (37.0%) and active search (25.9%). The positivity rate during the study period was 27.2% in Salvador and 11.5% in Rio de Janeiro. A total of 14.1% of cases were monitored, and most were asymptomatic (42.5%) or mild (52.3%). No deaths were reported among monitored cases. The proportion of fully vaccinated individuals was 91.8%. Contact tracing identified 25.1% of reported contacts, and 41.5% declined testing.

**CONCLUSIONS:**

The intervention facilitated expanded testing. Primary health care service units and active search were the strategies that reached more individuals for testing. Telemonitoring and contact tracing were the most challenging components to implement in primary health care services units and, given their importance, should be strengthened for future pandemics. These findings underscore the relevance of surveillance for assessing public health measures, identifying gaps, and supporting data-driven decision-making to improve epidemic management.

## INTRODUCTION

As a global emergency, Covid-19 has caused more than 775 million infections and more than 7 million deaths, mainly affecting populations under socioeconomic vulnerability^1,2^. Although Covid-19 is transitioning from a pandemic to an endemic disease, the World Health Organization (WHO) reported that at least 95 countries recorded Covid-19 cases in July 2024, and at least 31 countries reported Covid-19 deaths. Therefore, concerns remain about the emergence of new variants, new surges, and how to prepare for them^1^.

Brazil was severely affected by the Covid-19 pandemic, with more than 38.8 million cases and more than 700,000 deaths recorded by September 2, 2024^3^. In the absence of effective treatment, non-pharmaceutical interventions were key to limiting the pandemic; these strategies were focused on rapid diagnosis and prevention, making essential surveillance measures fundamental for mitigating the impact of Covid-19 and for its prevention^4^. Massive testing, contact tracing, quarantine of cases and contacts, and telemonitoring contributed to reducing the morbidity and mortality of Covid-19 and to avoiding the overburdening of health systems^5,6^. Surveillance data was fundamental for tracking transmission, evaluating the effectiveness of preventive measures, ensuring timely access to healthcare, and identifying when to intensify prevention strategies during peaks^7^.

Primary health care (PHC) is an approach that brings health services closer to the community, providing an equitable, inclusive, and cost-effective way to achieve universal health coverage^8^. In the Brazilian Unified Health System (*Sistema Único de Saúde* — SUS), PHC is the first point of contact with the health system and facilitates access to more specialized services^9^. During the Covid-19 pandemic, PHC services within SUS were prepared and trained to strengthen the health system's resilience in response to the crisis. The care flows in these services were reorganized, telemonitoring of positive cases and contacts was implemented, and active and passive surveillance was reinforced^9,10^.

PHC was also responsible for involving the community and promoting testing, vaccination, and other prevention methods^8,11^. In this context, demand creation is crucial for influencing behavior and increasing the uptake of Covid-19 testing and vaccination^12^. Demand creation refers to strategies aimed at encouraging people to seek health services by improving knowledge, attitudes, motivation, and decision-making, while directly addressing barriers through incentives, community campaigns, or counseling. These strategies are directed at the individual or community level and aim to increase demand for health services, products, or behaviors, not only to improve the availability or quality of the service itself^12,13^.

In 2022, the Covid-19 testing, isolation, quarantine, and telemonitoring (TQT Covid-19) study was launched at PHC units in neighborhoods under socioeconomic vulnerability in two Brazilian capital cities. The primary objective was to analyze the demand creation strategies used to reach and enroll individuals in Covid-19 testing and to describe the characteristics of the populations most reached by each strategy. The secondary objective was to describe the Covid-19 indicators used for the surveillance of TQT, and contact tracing in these vulnerable communities.

This study was guided by the following research questions: (1) Which demand creation strategies were most effective in reaching and engaging individuals in Covid-19 testing at PHC units, and what were the sociodemographic characteristics of the populations most reached by each strategy? and (2) What were the results of an intervention aimed at increasing testing and implementing monitoring, quarantine, and contact tracing?

## METHODS

### Study Design, Setting, and Participants

This cross-sectional study used surveillance data from the TQT Covid-19 study, which aimed to implement and expand testing, isolation, quarantine, e-health, and telemonitoring strategies for Covid-19 prevention at PHC services in neighborhoods under socioeconomical vulnerability in two large Brazilian capital cities: Rio de Janeiro (Rio de Janeiro State, Southeast Brazil) and Salvador (Bahia State, Northeast Brazil). The study protocol has been published elsewhere^14^.

The study area in Salvador was the *Cabula-Beirú* District, which has a population of 392,542 inhabitants across an area of 25.89 km^2^. The health district has 18 PHC units and 5 specialized units^15^. A total of 17 units were included in the study, covering 244,007 inhabitants. In Rio de Janeiro, the study was conducted in *Manguinhos*, a neighborhood located in the north zone that comprises 261.84 hectares and an estimated population of 40,586 inhabitants^16^. Two PHC units were selected for this study, covering a population of 38,954 as of July 2020^17^. The total population covered by the PHC units included in both cities was 282,961 individuals.

Teams composed of health professionals, research assistants, municipal health managers, and community-based health agents from selected PHC units were trained on the research protocol, the digital platform developed for the study, and an updates on the Covid-19 pandemic and safety measures^14^.

From July 2022 to July 2023, individuals who attended PHC units for antigen rapid diagnostic testing (Ag-RDT) were invited to participate in the study if they met the following criteria: presenting Covid-19 symptoms between three and seven days after symptom onset or having had close contact with a confirmed Covid-19 case. Symptomatic contacts were required to be between three and seven days after their own symptom onset, whereas asymptomatic contacts had to be between five and seven days after their last exposure. Eligibility was independent of prior vaccination or infection status. Individuals who agreed to participate completed a socio-behavioral questionnaire if they were aged 12 years old or older; for participants younger than 12 years, the questionnaire was completed by an accompanying parent or guardian. The socio-behavioral questionnaire was administered by research assistants or health professionals. Testing was conducted by a laboratory technician using either the Panbio™ Covid-19 Ag test (Abbott) or the Immuno-Rapid Covid-19 Ag test (WAMA Diagnostic). Participants who tested positive in the Ag-RDT were offered telehealth monitoring by a health professional, with follow-ups every 48 hours for those without comorbidities and every 24 hours for those with comorbidities until the end of isolation. Data were collected and recorded using a digital platform developed specifically for this project^18^. Participants were asked whether they had contact with anyone since the onset of symptoms or after receiving a positive test result. In case of an affirmative answer, authorization was requested to contact these individuals. Contact tracing was conducted through telehealth using telephone calls or text messages. When contacts were reached, they were invited to visit a PHC unit for testing.

### Ethical Issues and Consent

The TQT-Covid-19 study was conducted according to Brazilian research ethics regulations (Resolution CNS No. 466, Brazil, 2012; and Resolution CNS No. 510, Brazil, 2016) and international research ethics guidelines. The study was approved by the Ethics Research Committees of the World Health Organization (Protocol ID: CERC.0128A and CERC.0128B) and by local Brazilian Institutional Review Boards in Salvador (ISC/UFBA: 53844121.4.1001.5030) and Rio de Janeiro (INI/Fiocruz: 53844121.4.2001.5262; ENSP/Fiocruz: 53844121.4.3001.5240; and SMS/RJ: 53844121.4.3002.5279). All individuals invited to participate received a verbal explanation of the study's objectives and procedures, with the opportunity to ask questions before signing the informed consent. The form outlined the study's purpose, potential risks and benefits, the voluntary nature of participation, and the use of data from swab collection and laboratory test results. Participants aged 18 or older signed the consent form. For individuals under 18, consent was obtained from a parent or guardian, and adolescents aged 12 to 17 also signed an assent form. Participants could withdraw from the study at any time or skip questions they considered sensitive or distressing. To ensure confidentiality, all data were securely stored on a digital platform, and no personal identifiers were used in public presentations or publications.

### Variables Definition

Age was categorized into four groups: 12 to 25 years, 26 to 49 years, and 50 years or older. Gender was dichotomized into female (cisgender women and transgender women) and male (cisgender men and transgender men). Race was categorized into two groups: Black and Brown *versus* others, which included White, Asian, and Indigenous. Education levels were categorized as secondary education or less (ranging from no formal education to completion of high school) and higher education (any post-secondary education, whether completed or not). Monthly family income was categorized using two minimum wages as a cut-off. In 2022, the minimum wage in Brazil was BRL 1,212 (approximately USD 232, based on the 2022 average exchange rate of 1 USD ≈ 5.22 BRL). Participants were asked about the number of rooms in their homes and the number of individuals living there, allowing for the estimation of the density of people per room, which was categorized using a cut-off point of 0.5: low density (< 0.5), indicating good living conditions, and high density (> 0.5), indicating worse living conditions. Participants were asked to self-report diagnosed chronic comorbidities using a predefined list of specific chronic conditions by answering "yes" or "no" to each: obesity, diabetes mellitus, heart disease or high blood pressure, respiratory diseases (e.g., asthma, pulmonary emphysema, tuberculosis), current or past cancer treatment, hematological diseases (including sickle cell anemia), advanced-stage chronic kidney disease, chromosomal conditions associated with immune fragility (e.g., Down syndrome, Turner syndrome), liver diseases (e.g., fatty liver, hepatitis, cirrhosis), autoimmune diseases (e.g., systemic lupus erythematosus, rheumatoid arthritis, immune thrombocytopenia), immunodeficiencies (e.g., HIV infection, leukemia), or were asked to specify other conditions. Participants were then classified as having one or more chronic comorbidities *versus* none.

Definitions of Covid-19 suspected and confirmed cases, and contacts followed the WHO and Brazilian Ministry of Health criteria^19,20^. When testing positive, symptoms were classified as mild, moderate, severe, or critical following the classification of the Brazilian Ministry of Health guidelines^20^. The primary Covid-19 vaccination series was considered complete when participants reported receiving either a single-dose vaccine, such as the Janssen^®^ vaccine, or both doses of a two-dose vaccine, including CoronaVac, Pfizer or Oxford AstraZeneca. Quarantine compliance was assessed through telehealth; individuals were asked about having conditions to undergo 7 to 10 days of isolation without social gatherings.

Demand creation strategies that reached individuals were assessed with the question: "How did you hear about testing in this project?" Participants could choose one answer from 16 possible options, which were grouped into five categories: online strategies, traditional means, health care units, community, and active search. Online strategies included the project app, social media (Instagram, WhatsApp, Facebook, TikTok), informational websites, podcasts, and a chatbot. Traditional means included posters and flyers, local radio, television, newspaper, and a loudspeaker vehicle. Health care units’ strategies included individuals who were referred by health professionals at the PHC unit or individuals who passed by the unit and saw the study taking place. Community strategies included school, workplaces, religious institutions, other community institutions, and word of mouth among family members, neighbors, or friends. Active search referred to community health agents from the clinic or territory or health professionals responsible for contact tracing.

### Surveillance Indicators

Two groups of surveillance indicators were estimated. The first group focused on testing and prevention of Covid-19 and included the number of tests administered per site, the positivity rate, testing uptake, refusal of testing, and vaccination rates. Tests administered per site were estimated as the number of tests performed in relation to the covered population. The positivity rate was calculated as the number of positive Covid-19 tests divided by the total number of Ag-RDT administered. Covid-19 testing uptake was defined as the proportion of individuals tested among those who presented symptoms or were close contacts. Two indicators of vaccination were estimated. The first was the proportion of people vaccinated, defined as the number of individuals who received at least one dose divided by the total number of individuals who agreed to participate in the study. The second was the proportion of individuals who had completed the primary vaccination series, calculated as the number of individuals who complete the primary vaccination series divided by the total number of study participants. Covid-19 monthly incidence was also estimated by dividing the number of patients with positive results by the total population covered, multiplied by 10,000 inhabitants.

The second group of indicators was related to engagement strategies and management of cases among monitored patients and included telemonitoring, quarantine, and contact tracing indicators. The proportion of Covid-19 cases monitored was calculated by dividing the number of cases monitored by the total number of positive cases. The proportion of asymptomatic Covid-19 cases was calculated by dividing the number of asymptomatic cases by the total number of monitored cases. The proportion of mild Covid-19 cases was calculated by dividing the number of mild cases by the total number of monitored cases. Similarly, the proportion of moderate Covid-19 cases was determined by dividing the number of moderate cases by the total number of monitored cases. For severe cases, the proportion was obtained by dividing the number of severe cases by the total number of monitored cases. The proportion of Covid-19 cases referred to hospitals or specialized care was calculated by dividing the number of referrals by the total number of monitored cases. The Covid-19 fatality rate was defined as the proportion of deaths among all monitored Covid-19 cases. Compliance with isolation was assessed by calculating the proportion of monitored individuals who reported being able to comply with isolation for seven to ten days. The contacts traced indicator was defined as the number of contacts successfully traced by PHC units divided by the total number of contacts reported by the cases. Refusal of testing was calculated by dividing the number of contacts who declined testing by the total number of contacts traced.

### Study Size

The study aimed to increase the number of Covid-19 tests by 10% compared to the volume conducted during the six months preceding study initiation at each site. Accordingly, we estimated performing 12,000 tests.

### Statistical Analysis

To estimate the surveillance indicators, all available information from the general population attending PHC units for testing, as well as from individuals who agreed to participate in the study, was utilized. To analyze demand creation strategies, individuals aged 12 years old and older who completed the socio-behavioral questionnaire were included. Participants under 12 years were excluded to avoid data dependence, as their parents or guardians completed their questionnaires.

Descriptive statistics of population characteristics were conducted. In the bivariate analysis, the association between sociodemographic variables and each demand creation strategy was assessed using the chi-square test. Logistic regression models were fitted for each demand creation strategy, using all other strategies combined as the comparison group. Variables were selected based on statistical significance in the bivariate analysis (p < 0.05) and relevant literature. Multicollinearity was assessed by examining variance inflation factors (VIF), and no concerning levels were detected. Adjusted odds ratios (OR_a_) and corresponding 95% confidence intervals (95%CI) were estimated. To refine the models, a backward elimination approach was applied, with variable retention based on likelihood ratio tests. The Akaike Information Criterion and Bayesian Information Criterion were used to compare competing models and identify the most parsimonious with the best fit. Model performance was evaluated using the Hosmer–Lemeshow goodness-of-fit test. Data were analyzed using R software, version 4.2.2 (https://www.r-project.org), and Stata, version 15.0.

## RESULTS

The TQT Covid-19 study reached 12,883 individuals, comprising 8,437 from Salvador and 4,446 from Rio de Janeiro (Figure).

**Figure:**
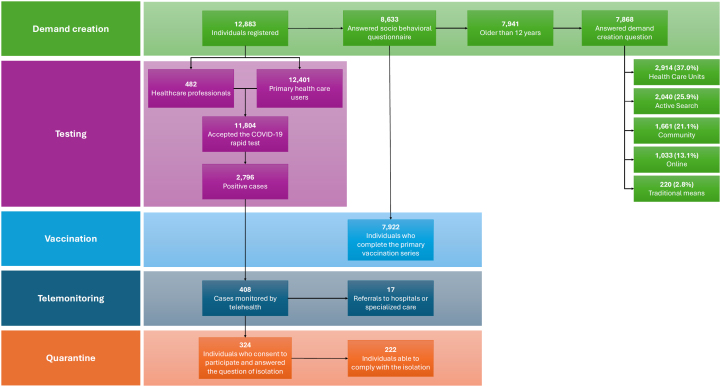
Flowchart of the Covid-19 Testing, Isolation, Quarantine, and Telemonitoring Study.

### Demand Creation Strategies

A total of 7,941 individuals older than 12 years answered the socio-behavioral questionnaire, and 7,868 who answered the demand creation question were included in this analysis. The characteristics of these individuals are presented in Table 1. The majority were female (69.7%), aged 26 to 49 years (52.6%), Black or Brown (84.0%), had completed high school or less (74.5%), and reported a monthly family income of fewer than two minimum wages (71.9%). The demand creation strategies that reached the largest proportion of the population were referrals from health care units (37.0%) and active search (25.9%), as shown in Table 2. In the multivariate analysis (Table 3), individuals aged 26 to 49 years (OR_a_ = 0.77; 95%CI 0.65–0.91) and individuals aged 50 years old or older (OR_a_ = 0.46; 95%CI 0.36–0.57) were significantly less likely to be reached by online strategies compared to those aged 12 to 25 years. However, individuals aged 26 to 49 years and those aged 50 years or older had higher chances of being reached by active search strategies (OR_a_ = 1.21; 95%CI 1.01–1.44 and OR_a_ = 1.28; 95%CI 1.05–1.56, respectively) compared to those aged 12 to 25 years. Females were more likely to be reached by health care unit strategies (OR_a_ = 1.29; 95%CI 1.13–1.47) but less likely by community strategies (OR_a_ = 0.83; 95%CI 0.71–0.97). Compared to Salvador, individuals from Rio de Janeiro had lower odds of being reached by online strategies (OR_a_ = 0.09; 95%CI 0.07–0.13), traditional means (OR_a_ = 0.29; 95%CI 0.18–0.45), and community strategies (OR_a_ = 0.32; 95%CI 0.27–0.39), but higher odds of being reached by health care units (OR_a_ = 2.65; 95%CI 2.34–3.00) and active search (OR_a_ = 1.80; 95%CI 1.57–2.06). Individuals with higher education had greater chances of being reached by online strategies (OR_a_ = 1.66; 95%CI 1.43–1.92) but lower chances of being reached by active search (OR_a_ = 0.65; 95%CI 0.54–0.78). Individuals with higher income had higher chances of being reached by community strategies (OR_a_ = 1.38; 95%CI 1.17–1.62) and lower chances of being reached by active search (OR_a_ = 0.64; 95%CI 0.54–0.75). Individuals living in worse conditions (density > 0.5) had lower chances of being reached by online strategies (OR_a_ = 0.83; 95%CI 0.72–0.96) but higher chances of being reached by active search (OR_a_ = 1.19; 95%CI 1.04–1.36). Individuals with chronic conditions were more likely to be reached by traditional means (OR_a_ = 1.54; 95%CI 1.17–2.04) and by health care unit strategies (OR_a_ = 1.12; 95%CI 0.99–1.28).

**Table 1:** Characteristics of participants included in the analysis, overall and by site. Covid-19 Testing, Isolation, Quarantine, and Telemonitoring Study.

Characteristic		n	Total	Salvador	Rio de Janeiro
				n = 5,776 (73.4%)	n = 2,092 (26.6%)
Age (years), n (%)					
	12 to 25 year	7,762	1,477 (19.0)	1,025 (17.9)	452 (22.1)
	26 to 49		4,088 (52.7)	3,100 (54.3)	988 (48.2)
	≥ 50		2,197 (28.3)	1,588 (27.8)	609 (29.7)
Gender, n (%)					
	Male	7,716	2,339 (30.3)	1,655 (29.2)	684 (33.4)
	Female		5,377 (69.7)	4,015 (70.8)	1,362 (66.6)
Race, n (%)					
	Black/Brown	7,761	6,522 (84.0)	5,129 (89.8)	1,393 (67.9)
	White/Asian/Indigenous		1,239 (16.0)	582 (10.2)	657 (32.0)
Schooling, n (%)					
	Secondary education or less	7,757	5,783 (74.5)	4,153 (72.7)	1,630 (79.5)
	Higher education		1,974 (25.4)	1,554 (27.2)	420 (20.5)
Monthly family income^a^, n (%)					
	Less than two minimal wages	4,973	3,578 (71.9)	2,436 (74.6)	1,142 (66.9)
	More than two minimal wages		1,395 (28.0)	829 (25.4)	566 (33.1)
Density of occupants per room, n (%)					
	Good living conditions (< 0.5)	7,864	3,698 (47.0)	2,944 (51.0)	754 (36.0)
	Worse living conditions (> 0.5)		4,166 (53.0)	2,828 (49.0)	1,348 (64.0)
Comorbidities, n (%)					
	None	7,715	5,359 (69.5)	3,977 (70.7)	1,382 (66.1)
	One or more		2,356 (30.5)	1,646 (29.3)	710 (33.9)

a The minimum wage in Brazil in 2022 was BRL 1,212 (approximately USD 232, based on the 2022 average exchange rate of 1 USD ≈ 5.22 BRL).

**Table 2:** Bivariate analysis of the association between sociodemographic characteristics and demand creation strategies in the Covid-19 Testing, Isolation, Quarantine, and Telemonitoring Study.

Characteristics		Demand creation strategies				
		Online strategies	Traditional means	Health Care Unit	Community	Active search
Total, n (%)		1,033 (13.1)	220 (2.8)	2,914 (37.0)	1,661 (21.1)	2,040 (25.9)
Age (years), n (%)						
	12 to 25	242 (16.4)^c^	34 (2.3)	527 (35.7)	308 (20.8)	366 (24.8)^b^
	26 to 49	596 (14.6)^c^	112 (2.7)	1,491 (36.5)	876 (21.4)	1,013 (24.8)^b^
	≥ 50	179 (8.1)^c^	74 (3.4)	853 (38.8)	462 (21.0)	629 (28.6)^b^
Gender, n (%)						
	Male	299 (12.7)	76 (3.2)	811 (34.4)^b^	540 (22.9)^b^	629 (26.7)
	Female	717 (13.3)	145 (2.7)	2,051 (38.0)^b^	1,100 (20.4)^b^	1,378 (25.6)
Race, n (%)						
	Black/Brown	111 (9.0)^c^	30 (2.4)	536 (43.3)^c^	229 (18.5)^b^	333 (26.9)
	White/Asian/Indigenous	904 (13.9)^c^	190 (2.9)	2,336 (35.8)^c^	1,417 (21.7)^b^	1,675 (25.7)
Site, n (%)						
	Salvador	993 (17.2)^c^	198 (3.4)^c^	1,735 (30.0)^c^	1,470 (25.4)^c^	1,380 (23.9)^c^
	Rio de Janeiro	40 (1.9)^c^	22 (1.0)^c^	1,179 (56.4)^c^	191 (9.1)^c^	660 (31.5)^c^
Schooling, n (%)						
	Secondary education or less	645 (11.1)^c^	163 (2.8)	2,180 (37.5)	1,207 (20.8)	1,613 (27.8)^c^
	Higher education	375 (9.0)^c^	58 (2.9)	698 (35.4)	444 (22.5)	399 (20.2)^c^
Monthly family income^a^, n (%)						
	Less than two minimal wages	425 (11.8)	110 (3.1)	1,377 (38.4)^b^	672 (18.7)^b^	1,001 (27.9)^c^
	More than two minimal wages	194 (13.9)	41 (2.9)	582 (41.7)^b^	314 (22.5)^b^	264 (18.9)^c^
Density of occupants per room, n (%)						
	Good living conditions (< 0.5)	556 (15.0)^c^	97 (2.6)	1,344 (36.3)	823 (22.3)^b^	878 (23.7)^c^
	Worse living conditions (> 0.5)	476 (11.4)^c^	123 (2.9)	1,568 (37.6)	837 (20.1)^b^	1,162 (27.9)^c^
Comorbidities, n (%)						
	None	794 (14.7)^c^	133 (2.5)^b^	1,925 (35.7)^c^	1,152 (21.4)^b^	1,384 (25.7)
	One or more	228 (9.7)^c^	85 (3.6)^b^	952 (40.4)^c^	452 (19.2)^b^	641 (27.2)

a The minimum wage in Brazil in 2022 was BRL 1,212 (approximately USD 232, based on the 2022 average exchange rate of 1 USD ≈ 5.22 BRL);

b < 0.05;

c < 0.001.

**Table 3:** Logistic regression of the association between sociodemographic characteristics and demand creation strategies in the Covid-19 Testing, Isolation, Quarantine, and Telemonitoring Study.

Characteristics		Demand creation strategies				
		Model 1	Model 2	Model 3	Model 4	Model 5
		Online strategies	Traditional means	Health Care Unit	Community	Active search
		OR_a_ (95%CI)	OR_a_ (95%CI)	OR_a_ (95%CI)	OR_a_ (95%CI)	OR_a_ (95%CI)
Age (years)						
	12 to 25	1	-	-	-	1
	26 to 49	0.74 (0.62–0.87)	-	-	-	1.21 (1.01–1.44)
	≥ 50	0.42 (0.34–0.51)	-	-	-	1.28 (1.05–1.56)
Gender						
	Male	-	-	1	1	-
	Female	-	-	1.29 (1.13–1.47)	0.83 (0.71–0.97)	-
Site						
	Salvador	1	1	1	1	1
	Rio de Janeiro	0.09 (0.07–0.13)	0.29 (0.18–0.45)	2.65 (2.34–3.00)	0.32 (0.27–0.39)	1.80 (1.57–2.06)
Schooling						
	Secondary education or less	1	-	-	-	1
	Higher education	1.66 (1.43–1.92)	-	-	-	0.65 (0.54–0.78)
Monthly family income^a^						
	Less than two minimal wages	-	-	1	1	1
	More than two minimal wages	-	-	1.07 (0.93–1.23)	1.38 (1.17–1.62)	0.64 (0.54–0.75)
Density of occupants per room						
	Low density (< 0.5)	1	-	-	-	1
	High density (≥ 0.5)	0.83 (0.72–0.96)	-	-	-	1.19 (1.04–1.36)
Comorbidities						
	None	-	1	1	1	-
	One or more	-	1.54 (1.17–2.04)	1.12 (0.99–1.28)	0.94 (0.80–1.10)	-

a The minimum wage in Brazil in 2022 was BRL 1,212 (approximately USD 232, based on the 2022 average exchange rate of 1 USD ≈ 5.22 BRL).

OR_a_: adjusted odds ratio; 95%CI: 95% confidence interval. Note: a dash (-) indicates that the variable was not included in the model; Hosmer–Lemeshow goodness-of-fit test: Model 1 = 0.91; Model 2 = 0.43; Model 3 = 0.24; Model 4 = 0.22; Model 5 = 0.27.

### Surveillance Indicators

#### Testing

During the study, 11,804 Ag-RDT were performed: 7,863 in Salvador and 3,941 in Rio de Janeiro. Most people reached by the study (91.6%) were tested. Considering the population covered by PHC units, Rio de Janeiro (10.1%) had a higher testing rate compared to Salvador (3.2%). The positivity rate was higher in Salvador (29.3%) than in Rio de Janeiro (12.5%) (Table 4).

**Table 4:** Surveillance indicators of the Covid-19 Testing, Isolation, Quarantine, and Telemonitoring Study.

Indicators		Name	Definition	Total				Salvador			Rio de Janeiro	
				Numerator	Denominator	Result (%)	Numerator	Denominator	Result (%)	Numerator	Denominator	Result (%)
Testing and prevention of Covid-19	Testing	Tests administered per site	Number of Ag-RDT performed by the covered population	11,804	282,961	4.2	7,863	244,007	3.2	3,941	38,954	10.1
		Positivity rate	Number of positive Covid-19 cases divided by the total number of Ag-RDT applied	2,796	11,804	23.7	2,303	7,863	29.3	493	3,941	12.5
		Covid-19 testing uptake	Proportion of individuals tested among those who presented symptoms or were close contacts	11,804	12,883	91.6	7,863	8,437	93.1	3,941	4,446	88.6
	Vaccination	Vaccination uptake	Proportion of study participants with at least one Covid-19 vaccine dose	8,273	8,626	95.9	5,987	6,197	96.6	2,286	2,429	94.1
		Primary vaccination series completed	Proportion of study participants who complete the Covid-19 primary vaccination series	7,922	8,619	91.9	5,754	6,192	92.9	2,168	2,427	89.3
Engagement strategies with healthcare units	Telemonitoring	Proportion of positive cases monitored	Number of Covid-19 cases monitored divided by the total number of positive cases	408	2,796	14.6	344	2,303	14.9	64	493	13.0
		Proportion of asymptomatic Covid-19 cases	Number of asymptomatic Covid-19 cases divided by the total number of monitored cases	135	400	33.7	102	336	30.4	33	64	51.6
		Proportion of mild Covid-19 cases	Number of mild Covid-19 cases divided by the total number of monitored cases	241	400	60.2	218	336	64.9	23	64	35.9
		Proportion of moderate Covid-19 cases	Number of moderate Covid-19 cases divided by the total number of monitored cases	22	400	5.5	16	336	4.8	6	64	9.4
		Proportion of severe Covid-19 cases	Number of severe Covid-19 cases divided by the total number of monitored cases	2	400	0.5	0	336	0	2	64	3.1
		Proportion of referrals to hospitals or specialized care	Cases referred to hospitals or specialized care divided by the total number of monitored cases	17	399	4.3	15	335	4.5	2	64	3.1
		Covid-19 fatality rate	Proportion of deaths among all monitored Covid-19 cases.	0	408	0	0	344	0	0	64	0.0
	Quarantine	Compliance with isolation	Proportion of monitored individuals who reported being able to comply with isolation	222	324	68.5	194	261	74.3	28	63	44.4
	Contact tracing	Contacts traced	Number of contacts successfully traced by PHC units divided by the total number of contacts reported by the cases	-	-	-	195	777	25.1	-	-	-
		Refusal of testing	Number of contacts who declined testing divided by the total number of contacts traced	-	-	-	81	195	41.5	-	-	-

PHC: primary health care; Ag-RDT: antigen rapid diagnostic test.

Note: Among the 344 individuals monitored in Salvador, eight were not classified by severity level due to missing data, nine had missing information regarding referrals, and 24 had missing data for the isolation question.

#### Incidence

In Salvador, tests were performed from July 2022 to March 2023. The months with the highest Covid-19 incidence were July and November 2022, with 66.0 and 70.9 cases per 10,000 inhabitants, respectively. In Rio de Janeiro, tests were performed from November 2022 to July 2023, and the month with the highest incidence was November 2022, with 108.0 cases per 10,000 inhabitants (Table 5).

**Table 5:** Covid-19 incidence per month in the Covid-19 Testing, Isolation, Quarantine, and Telemonitoring Study.

Year	Month	Salvador			Rio de Janeiro		
		Number of positive cases	Population covered by PHC	Incidence (per 10,000)	Number of positive cases	Population covered by PHC	Incidence (per 10,000)
2022	July	279	42,301	66.0	-	-	-
	August	77	190,453	4.0	-	-	-
	September	4	215,033	0.2	-	-	-
	October	2	244,007	0.1	-	-	-
	November	1,731	244,007	70.9	249	23,062	108.0
	December	305	244,007	12.5	47	23,062	20.4
2023	January	0	201,706	0.0	5	23,062	2.2
	February	2	53,554	0.4	34	38,954	8.7
	March	0	28,974	0.0	123	38,954	31.6
	April	-	-	-	27	38,954	6.9
	May	-	-	-	18	38,954	4.6
	June	-	-	-	4	38,954	1.0
	July	-	-	-	3	38,954	0.8

PHC: primary health care.

#### Vaccination

Among individuals who reported their Covid-19 vaccination status, 95.9% indicated receiving at least one dose, and 91.9% had completed the primary vaccination series. Completion was higher in Salvador (92.9%) than in Rio de Janeiro (89.3%) (Table 5).

#### Telemonitoring

A total of 408 cases were monitored by telehealth in PHC units, representing 14.6% of participants for whom monitoring was offered: 344 (14.9%) in Salvador and 64 (13.0%) in Rio de Janeiro. Most monitored cases were mild in Salvador (64.9%) and asymptomatic in Rio de Janeiro (51.6%). Severe cases represented 3.2% in Rio de Janeiro and none in Salvador. No deaths were recorded monitored cases (Table 4). Cases referred to hospitals or specialized services were 4.5% in Salvador and 3.1% in Rio de Janeiro.

#### Quarantine

Participants who reported being able to undergo isolation were 74.3% in Salvador and 44.4% in Rio de Janeiro (Table 4).

#### Contact Tracing

Contact tracing was performed only in Salvador. Cases reported 777 contacts, and one fourth were traced by PHC professionals (25.1%). Among those traced, 41.5% refused testing (Table 4).

## DISCUSSION

Our results indicate that PHC units and active search were successful in engaging a large number of individuals for testing. However, each demand creation strategy demonstrated varying degrees of success across demographic groups. Vulnerable populations — older adults, individuals with low educational attainment, low family income, and poor living conditions — were reached more effectively through active search by community health agents. Referrals from PHC units were common among women and individuals with chronic conditions. Online strategies were particularly effective in reaching younger individuals, those with higher education, and those with better living conditions. Community strategies were sources of information for men and those with higher income. Few individuals accessed information through traditional means, which was significant only for individuals with chronic conditions. These findings suggest that demand creation strategies should be tailored to the demographic and socioeconomic characteristics of the target population.

Our indicators showed the extent of the TQT-Covid-19 study, which involved more than 12,000 people reached by the intervention in PHC units. Testing indicators demonstrated high uptake. As expected, Covid-19 incidence and prevalence during the study period were lower than in earlier studies conducted at the onset of the pandemic, when vaccination was not yet available^16,17^. Notably, a peak occurred in November 2022 in both cities, coinciding with the outbreak of the Omicron variant, which spread globally in late 2022^21^. Most cases were asymptomatic or mild, and full vaccination coverage was comparable to national levels at that time^22,23^. Telemonitoring and contact tracing were the most compromised stages of surveillance, as not all positive cases and contacts could be followed. Challenges included lack of infrastructure, absence of internet and mobile devices in health services, and insufficient staff to manage case volume. Telemonitoring and contact tracing are not fully structured in PHC units within SUS, remaining areas for strengthening before future pandemics.

We observed high testing uptake, with more than half of traced contacts accepting testing. Previous studies indicate that social solidarity and perceived community-level benefits may motivate testing participation even without direct individual medical benefit^24,25^.

PHC units and active search showed greater effectiveness in reaching individuals in Rio de Janeiro, potentially reflecting stronger health system capacity, particularly at the PHC level. In contrast, lower effectiveness in Salvador may be attributable to structural limitations in the local health system^26^.

During the Covid-19 pandemic, telehealth expanded considerably due to high demand for healthcare and the need for social distancing. A critical barrier, however, was unequal access and implementation^27^. Online strategies for demand creation primarily reached individuals with access to digital platforms and basic digital literacy. This reflects the digital divide, rooted in structural barriers widely identified in Brazil^28^. The same pattern likely influenced telemonitoring implementation. We observed low adherence to telemonitoring, which may be attributed not only to the lack of infrastructure in health services but also to the population's limited access to cellphones, computers, and stable, high-quality internet connections. This highlights the challenges to achieving health equity in telehealth, a critical consideration in planning for the scale-up of telehealth for future pandemics.

Results of a study that modeled the transmission of SARS-CoV-2 in different scenarios showed that contact tracing using manual methods was insufficient to contain transmission, as delays between confirming a case and reaching its contacts may undermine the effectiveness of contact tracing in mitigating the spread of the epidemic^29^. These delays are expected, as observed in our indicators, with more than three-quarters of contacts not even located. The experience of high-income countries has shown that using digital tools that alert contacts in real time, or as soon as a case is confirmed, achieves better performance in epidemic control, reducing the effective reproduction number of Covid-19 infections and deaths^30,31^. However, the introduction of digital tools for pandemic response is challenging for low- and middle-income countries^32^. In preparing for future pandemics, implementing digital tools for contact tracing is crucial, while addressing ethical and data protection issues^29,31^. Additionally, such tools must be easily accessible to the community and will require strategies to engage older adults, individuals with lower socioeconomic status, and those with low digital literacy.

We acknowledge some limitations in the study. The effectiveness of the proposed intervention was not estimated, nor was the adequacy of local resources to ensure proper monitoring of Covid-19 cases. Additionally, demand creation activities were not compared across cities, which may have influenced the observed differences in the effectiveness of strategies in each location. Contact tracing data were also limited due to challenges in identifying and following up contacts. In many cases, individuals who should have been identified as contacts were entered into the system as primary cases. This misclassification may have compromised the accuracy of related indicators and hindered the effectiveness of epidemic control. These limitations stem from inadequate resources for effective contact tracing, a shortage of trained professionals, and limited experience in managing large-scale efforts during a pandemic. Despite these limitations, the indicators demonstrated the role of PHC in the pandemic response as a cornerstone for decentralized and equitable health access. Notably, many of the limitations identified lie beyond the primary objectives of this study. However, future research should incorporate longitudinal assessments of similar interventions and include comparable data across different cities to enhance cross-site analysis. A qualitative evaluation of the telemonitoring process would also be valuable in identifying the main barriers to its success.

In conclusion, demand creation strategies must be tailored to the specific population profiles they aim to reach, as different approaches are required to engage various groups. Surveillance was essential for understanding the response to the pandemic, allowing for comparison with other scenarios, identifying the interventions with the greatest impact, determining the population reached, and assessing the effectiveness of these interventions over time. PHC played a critical role in implementing testing, telemonitoring, and contact tracing strategies during pandemics; however, there is a need to strength the health system capacity to ensure it is adequately prepared to respond effectively to future public health crises. This includes leveraging technology and digital health tools for telehealth and contact tracing by developing accessible and inclusive initiatives, especially in low- and middle-income countries. Surveillance indicators can provide valuable insights for managing health services, potentially informing decision-making and supporting the planning of context-specific control measures. Although further validation is required, these findings could help guide more timely and targeted responses to emerging needs in future public health emergencies.

## Data Availability

Data for this study are available upon request.
